# U-Net-based reactive center loop-identifier for serpins

**DOI:** 10.1093/bioadv/vbag132

**Published:** 2026-05-07

**Authors:** Jason Liang, Chloe Wang, Lei Zhou

**Affiliations:** L&L Biotechnology, Gainesville, FL 32601, United States; L&L Biotechnology, Gainesville, FL 32601, United States; Department of Molecular Genetics and Microbiology, College of Medicine, University of Florida, Gainesville, FL 32610, United States

## Abstract

**Motivation:**

Serine protease inhibitors (serpins) are a large family of proteins conserved in all animals. A prominent enigma about serpins is that, while members of this family share an archetypal folding scheme, individual proteins have diverse functions and are involved in a variety of biological processes, including controlling inflammation, maintaining hormone homeostasis, and regulating osteogenesis. The reactive center loop (RCL) region plays a crucial role in determining the functional specificity of serpins. Yet, of the >48 000 serpins recorded in the UniProt database, only 78 have the RCL annotated. This is because the RCL, due to its high variability, cannot be identified using standard motif-matrix-based annotation strategies.

**Results:**

To overcome this deficiency, we tested neural network-based approaches for automatic RCL annotation. Using an expert-annotated training/validation dataset, we tested combinations of encodings and model architectures. U-Net-based models stood out as the best approach for this task. On the independent test dataset, U-Net-based models achieved ∼98% accuracy in identifying the RCL at the per-sequence level.

**Availability and implementation:**

Source code, training and testing datasets, and models are freely available at https://github.com/leizhou69/RCL-identifier or https://github.com/JasonLiang19/RCL-identifier under the MIT license.

## 1 Introduction

Serine protease inhibitors (serpins) are a large family of proteins conserved across all multicellular eukaryotes (Gettins 2002, Law *et al.* 2006, Gatto *et al.* 2013). The human genome has 36 serpin genes. Contrary to their family name, some “serpin” proteins do not function as protease inhibitors. For instance, human SerpinA6 and SerpinA7 genes encode corticosteroid-binding globulin (CBG) and thyroid hormone-binding globulin (TBG), respectively. To differentiate them from the bona fide protease-inhibitory serpins, these are often referred to as non-inhibitory serpins.

Mutations of various serpin genes are the underlying cause of distinct genetic diseases. For instance, mutations in the SerpinA1 gene cause life-threatening inflammation in the liver and lungs, affecting ∼1 in every 2500 people of European ancestry (Alpha-1 Foundation n.d.). Mutations in SerpinF1, on the other hand, are the underlying cause of osteogenesis imperfecta VI (Becker *et al.* 2011). In addition to being the underlying causes of a variety of genetic diseases, misregulations of specific serpin genes are implicated in cancer metastasis (Croucher *et al.* 2008) and neurodegenerative diseases (Crist *et al.* 2021, Zattoni *et al.* 2022).

**Figure 1:**
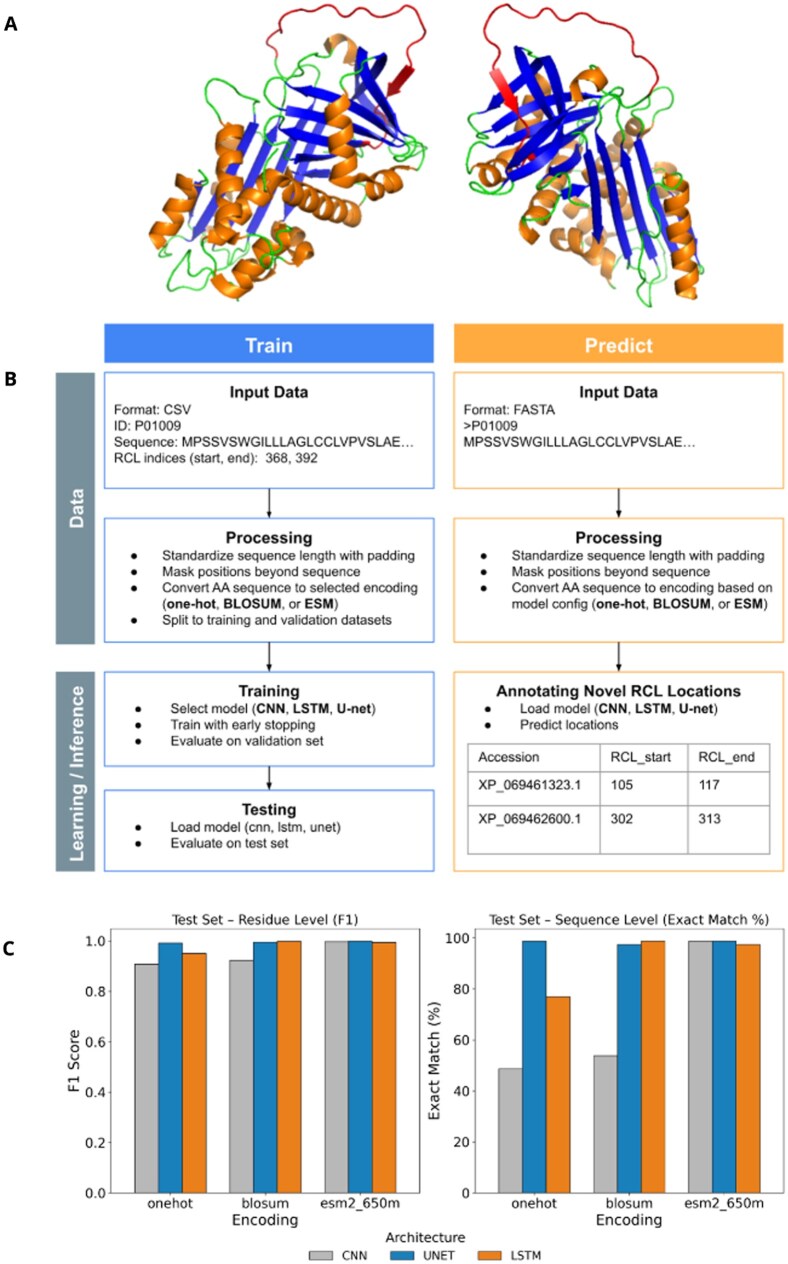
(A) 3D structure of a typical serpin (PDB 1qlp). RCL is the protruding loop (red) above the core globulin fold. (B) Training and prediction process. (C) Comparison of encoding and model architecture on the prediction performance at the residue and sequence level. More comparison in Table 3, available as supplementary data at *Bioinformatics Advances* online.

Serpin proteins are readily identifiable based on the presence of the serpin domain (IPR023796), which forms an archetypal structure of 3 β-sheets and 8–9 α-helixes (Fig. 1A) (Heit *et al.* 2013, Spence *et al.* 2021). An enigma concerning this group of proteins is how this shared folding scheme is adapted to endow rather diverse functions (Silverman *et al.* 2001, Whisstock *et al.* 2010). Different hypotheses have been put forward to explain this contradiction, such as the metastable nature of the serpin folding scheme and the impact of the reactive center loop (RCL) on protein conformational changes (Huntington 2011).

The RCL is a solvent-exposed stretch that protrudes out of the serpin scaffold (Fig. 1A). It is highly variable in terms of length and amino acid composition. For inhibitory serpins, the RCL mediates the interaction between the inhibitor and the target serine proteases, thus dictating target specificity (Huntington 2011). For non-inhibitory serpins, this region also exerts a regulatory role in the conformation of the globulin (Carrell *et al.* 2011). Yet, due to their highly variable lengths and little overall sequence similarity, RCLs cannot be identified using standard position-matrix-based approaches. Consequently, only 78 entries of the over 48 000 serpin proteins in the UniProt database have their RCL location annotated. The length of these annotated RCL regions in UniProt varies from 20 to 28 amino acids.

The lack of RCL annotations in most serpins prevents systematic comparison and analysis of this important class of proteins. We set out to test whether neural network-based strategies can fill this gap. Specifically, we considered (i) a basic convolution neural network (CNN), (ii) the U-Net, which was initially developed for biomedical image segmentation (Ronneberger *et al.* 2015) and has been extended successfully for predicting disordered regions in protein sequences (Kotowski *et al.* 2025), and (iii) Long short-term memory (LSTM), a recurrent neural network focused on long-range dependency.

## 2 Methods

### 2.1 Training/validation dataset

We selected over 20 genomes which represent the major branches of the animal kingdom and have relatively complete genome projects. All serpin proteins encoded by these genomes were batch-downloaded from UniProt. Those that already have an RCL annotation were set aside for the test dataset. For the majority that do not have RCL identification, we meticulously annotated the RCL regions using publicly available 3D structural information. The precise locations of RCL were determined following the methodology described by Li *et al.* (2018). A total of 1384 RCL regions were identified with sufficient confidence by the trained experts (Table 1, available as supplementary data at *Bioinformatics Advances* online). To form the negative dataset, we randomly extracted 2048 non-serpins from UniProt, matching the length distribution of the positive dataset. Together, this formed our training/validation dataset. All training/validation was based on a 0.8:0.2 split.

### 2.2 Independent test dataset

The 78 serpins in UniProt with an RCL annotation were used as the test dataset (Table 2, available as supplementary data at *Bioinformatics Advances* online). The negative test dataset consists of 1024 non-serpin proteins of similar length randomly extracted from UniProt. Any non-serpin proteins used in the training/validation dataset were excluded from the test dataset.

### 2.3 Encoding schemes

Three embedding methods were implemented: one-hot encoding, BLOSUM62 substitution matrix-based encoding, or the ESM2 encoding with the ESM2_650M model (Rives *et al.* 2021). Protein sequences were truncated or padded with zeros to a fixed length of 1024 residues. Each protein was represented as a tensor of shape (1024, D), where D is the embedding/feature dimension.

### 2.4 Model architecture

We evaluated three neural network architectures—CNN, U-Net, and LSTM—to compare performance. For all models, the input was comprised of encoded protein sequences with shape (batch_size, sequence_length, feature_dim). The output had shape (batch_size, sequence_length, 2), where the final dimension contains two logits representing the unnormalized scores for the RCL and non-RCL classes. Model hyperparameters were configurable via a configuration file; the descriptions below reflect the settings used for inference.

A 1D convolutional neural network (CNN) was implemented for per-residue classification. The model comprises four convolutional blocks, each containing a 1D convolution, batch normalization, ReLU activation, and dropout (*P* = .3). These blocks progressively extract sequence features and produce per-residue class logits.

The 1D U-Net architecture extends the CNN with an encoder–decoder design to preserve fine-grained positional information while learning contextual features. The encoder consists of four double-convolution (“DoubleConv”) blocks, with downsampling that halves the sequence length at each level. The bottleneck comprises an additional DoubleConv block. The decoder upsamples back to the original sequence length in four stages and integrates encoder features via skip connections and attention gates. A dropout rate of *P* = .25 was used, following DisorderUnetLM (Kotowski *et al.* 2025).

We also implemented a bidirectional LSTM to assess the contribution of long-range dependencies. Input embeddings are processed in both forward and reverse directions, maintaining recurrent cell states. Hidden states from both directions are concatenated, followed by dropout (*P* = .3) and a classification layer to produce per-residue logits.

### 2.5 Model training

All models were trained for up to 50 epochs using the Adam optimizer (learning rate = 0.001), with early stopping applied when the validation F1 score failed to improve for five consecutive epochs. Batch size was set to 32 for One-hot or BLOSUM encoded data, and to 4 for ESM2 embeddings due to GPU memory constraints. We used a masked binary cross-entropy loss to exclude padded/masked positions from the loss computation.

Training and inference were conducted on the University of Florida HiPerGator supercomputer using one or two Nvidia B200 GPUs.

## 3 Results

Of the three encoding schemes, ESM2 embeddings consistently achieved the strongest performance (Fig. 1C, Table 3, available as supplementary data at *Bioinformatics Advances* online). However, this comes at substantially higher computational cost during embedding generation, which can make large-scale annotation—especially for very large datasets or unusually long proteins—prohibitively expensive.

Using the simpler embeddings (One-hot and Blosum), U-Net and LSTM consistently outperformed the CNN, supporting our hypothesis that long-range contextual information is important for identifying RCL regions in serpin sequences. Notably, with ESM2 embeddings, even the CNN achieved strong performance (Fig. 1C; Table 3, available as supplementary data at *Bioinformatics Advances* online), likely because ESM2 already captures intra-sequence context during embedding. Consistent with this interpretation, models trained with ESM2 reached their minimum loss within ∼5 epochs, whereas models trained with One-hot or BLOSUM typically required >20 epochs.

At the residue level, the U-Net with ESM2 embeddings achieved the best overall metrics across all model-encoding combinations, including F1 = 0.9995, MCC = 0.9990, and accuracy = 0.9999 (Fig. 1C, Table 3, available as supplementary data at *Bioinformatics Advances* online). At the per-sequence level, the exact match rate is around 97%–98% for both U-Net and LSTM, indicating that for most of the sequences in the test dataset, the RCL identified by RCL-ID matched perfectly with those annotated by UniProt.

This high accuracy was somewhat surprising. We examined our strategy and implementation to ensure neither is susceptible to overfitting. However, since the target domain is a single protein family, implicit similarity between the training and testing datasets is unavoidable. To mitigate this, we threshold the training set to either 70% or 40% identity. We found that thresholding it by overall sequence identity did not significantly affect prediction accuracy (Table 4, available as supplementary data at *Bioinformatics Advances* online).

Through these tests, the performance of U-Net and LSTM was comparable, except when One-hot encoding was used, in which case U-Net performed much better (Fig. 1C). Compared to the LSTM, the U-Net trained ∼5× faster per epoch, converged in fewer epochs, and enabled faster inference. Accordingly, we choose U-Net as the default architecture for the identifier and provide three inference-ready models, onehot-Unet, Blosum-Unet, and ESM2-Unet.

## 4 Significance

The primary significance of the RCL-ID is that it enables systematic, large-scale (and ultimately systems-level) analyses of serpins. As described in the Introduction, serpin proteins share a conserved, archetypal fold yet exhibit highly diverse functions. Moreover, serpin gene copy number varies substantially across species. For example, the laboratory mouse (*Mus musculus*) has ∼60 protein-coding serpin genes, whereas the laboratory rat (*Rattus norvegicus*) has ∼50. The evolutionary forces driving these rapid changes in serpin repertoire size remain unclear, as does whether—and how—changes in the structure and function of individual serpins accompany expansion or contraction of the family.

Serpins also play critical roles in development and innate immunity in insects (Garrett *et al.* 2009, Benbow *et al.* 2019). A plethora of hypotheses have been postulated concerning how serpins may regulate immunity (Gatto *et al.* 2013, Bo and Zhao 2025). Interestingly, the impact of human SerpinA1 on inflammation and aging is recapitulated when it is expressed in transgenic Drosophila (Yuan *et al.* 2018). Yet, of the 28 serpin genes in the fruit fly, it is impossible to pinpoint which one is a true functional ortholog of human SerpinA1 using alignment-based phylogenetic analysis. Similarly, both human SerpinB8 and Drosophila SP42Da can inhibit furin and functionally complement one another (Dahlen *et al.* 1998, Ois Jean *et al.* 1998, Richer *et al.* 2004), yet typical phylogenetic analyses fails to identify them as a pair of orthologs. Together, these observations highlight the limitations of standard sequence alignment and position-specific matrix-based approaches in deriving a coherent, systems-level understanding of serpin functions across species.

Because the RCL is central to serpin structure and function, automatic RCL annotation provides a small but essential step toward enabling systematic comparative analyses and building a systems-level framework for interpreting serpin evolution. In turn, such a framework is foundational for understanding how specific human serpin alleles cause genetic disease or alter disease susceptibility.

## 5 Known limitations

Our training and validation datasets were annotated using high-confidence (AlphaFold) protein structures. However, for many serpin entries in the genomes we surveyed, AlphaFold does not provide high-quality predicted structures. As a result, our dataset is biased toward well-studied organisms with stronger structural coverage. The applicability toward out-of-distribution sequences from species that have not been subjected to genome sequencing remains to be tested.

When we applied the Blosum-Unet model to whole-genome protein sets, it occasionally identified RCL-like regions in non-serpin proteins. Therefore, the model is not suitable as a standalone tool for serpin discovery. Instead, it should be used after serpin proteins have been identified using established motif- or domain-based approaches, which are straightforward and widely used. We highlighted this limitation in the README file on the git repository.

For the ESM2-UNet model, sequences must be pre-embedded, which increases computational cost and can be limiting for very large datasets or unusually long proteins.

## Supplementary Material

vbag132_Supplementary_Data

## Data Availability

Source code, training and testing datasets, and models are freely available at https://github.com/leizhou69/RCL-identifier or https://github.com/JasonLiang19/RCL-identifier under the MIT license.
